# Supplementary material for: Development of a Prognostic Model of Overall Survival for Metastatic Hormone-Naïve Prostate Cancer in Japanese Men
Source: Cancers (Basel). 2022 Oct 2;14(19):4822. doi: 10.3390/cancers14194822 (PMC9563582; doi:10.3390/cancers14194822)
Supplement: Supplementary file 1 [file cancers-14-04822-s001.zip › cancers-1888496-supplementary.pdf]

Table S1. Univariate and multivariable analysis of prognostic factors for cancer specific survival

Variables	Univariate				Multivariable			
	95% CI				95% CI			
	p value	HR	Lower	Upper	p value	HR	Lower	Upper
Age <70 vs. ≧70 (years)	0.93	0.98	0.61	1.57	0.46	1.40	0.58	3.38
Include GS5	<0.001	3.29	1.95	5.57	0.066	2.57	0.94	7.03
PSA <200 vs. ≧200 (ng/mL)	0.38	1.23	0.77	1.95	0.96	0.97	0.28	3.41
BSI <1.5 vs. ≧1.5	0.14	1.61	0.86	3.04	0.18	2.20	0.69	7.01
Visceral metastasis	0.51	1.22	0.68	2.20	0.092	0.19	0.027	1.31
CRP <1.0 vs. ≧1.0 (mg/dL)	0.94	1.03	0.52	2.02	0.35	0.58	0.19	1.80
NLR <2.5 vs. ≧2.5	0.32	1.42	0.71	2.82	0.16	2.04	0.76	5.53
Hb <12 vs. ≧12 (g/dL)	0.86	1.07	0.51	2.24	0.65	0.77	0.25	2.38
ALP <300 vs. ≧300 (IU/L)	0.33	1.36	0.73	2.53	0.13	0.40	0.12	1.33
LDH <300 vs. ≧300 (IU/L)	<0.001	7.82	3.71	16.48	0.002	10.14	2.36	43.61

GP5 and LDH ≥ 300 were risk factors in univariate analysis. On the other hand, BSI ≥ 1.5 was not a risk factor. Multivariate analysis for CSS showed that only LDH ≥ 300 was an independent risk factor.

PSA; Prostate specific antigen, GP; Gleason Pattern, BSI; Bone scan index, CRP; C-reactive protein, NLR; Neutrophil-to-lymphocyte ratio, Hb; Hemoglobin, ALP; Alkaline phosphatase, LDH; Lactate dehydrogenase

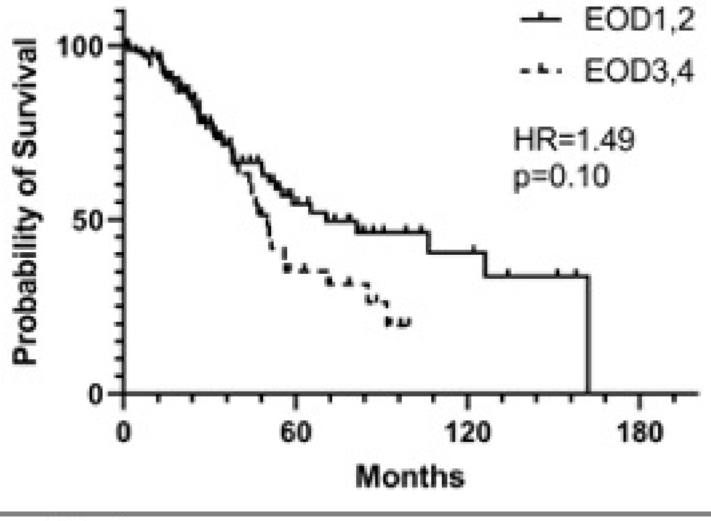

	Median OS	Numbers at risk		
EOD 1, 2	70.57	79	24	7
EOD 3, 4	50.17	53	10	0

Figure S1. Kaplan-Meier showing the difference in OS stratified by low EOD score (score 1,2) and high EOD score (score 3,4).

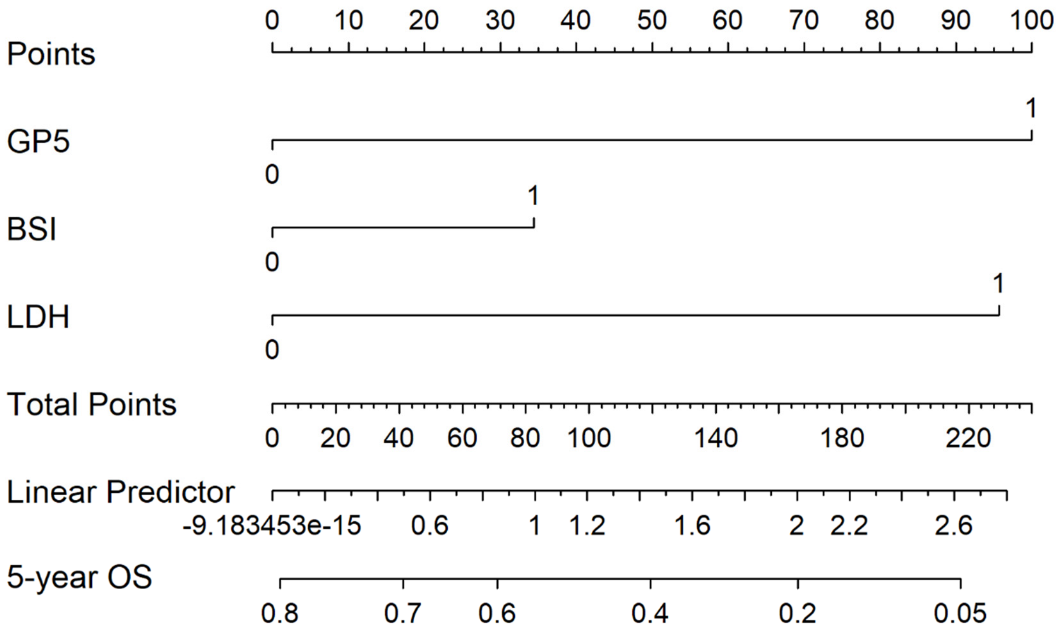

Figure S2. A nomogram was developed that combined the significant independent clinical variables.
